# Supplementary material for: Mathematical operations and equation solving with reconfigurable metadevices
Source: Light Sci Appl. 2022 Sep 7;11:263. doi: 10.1038/s41377-022-00950-1 (PMC9452564; doi:10.1038/s41377-022-00950-1)
Supplement: Supplementary file 1 — Supplementary Information [file 41377_2022_950_MOESM1_ESM.pdf]

Supplementary information for Mathematical Operations and Equation Solving with Reconfigurable Metadevices

Dimitrios C. Tzarouchis[1], Mario Junior Mencagli[2], Brian Edwards[1], and Nader Engheta[1]

[1]University of Pennsylvania, Department of Electrical and Systems Engineering, 19104, Philadelphia, USA
[2]University of North Carolina at Charlotte, Department of Electrical and Computer Engineering, Charlotte, NC, 28223, USA

ABSTRACT

Here we provide a series of supplementary information supporting the results and the discussion of the main text.

1 System simulations

In this section we describe the required elements for performing the system simulations used in obtaining the results appearing in the main text. We begin with a description of the basic computing module (MZI), a description of the feedback coupler, and then proceed to their assembly to form the equation solving platforms using the Miller and the Direct-Complex-Matrix (DCM) architectures.

1.1 The basic module

Both architectures were simulated using AWR Microwave Office ®[1], an RF circuit design software that allows the simulation of basic RF components such as power splitters, phase shifters, amplifiers etc.

The basic module that is used throughout the analysis is the MZI constructed as shown in Fig. (S1). This module consists of two 3 dB directional couplers and two phase shifters. The MZIs can be assembled and configured in a variety of ways to perform matrix multiplication, i.e. the Miller and DCM architectures. Although in our simulations we used the 1 GHz frequency, this choice is arbitrary and obviously, depending on the MZI platforms, other frequencies can be used.

As seen in Fig. (S1) the module was simulated as a combination of two 3 dB couplers (QHYB) and two phase shifters (PHASE), all centered to operate at 1 GHz. The QHYB coupling module is a typical symmetrical directional coupler as the one introduced in[2].

In the case of the multiplier module we used an MZI module followed by an amplifier, as can be seen in Fig. (S2). This module is the main building block for the DCM architecture.

1.2 Feedback coupler

Equation solving is enabled by routing the output of the matrix multiplication stage back toward the input (i.e. feedback). However, a system input must be provided and the output must be probed, all while perturbing the feedback as little as

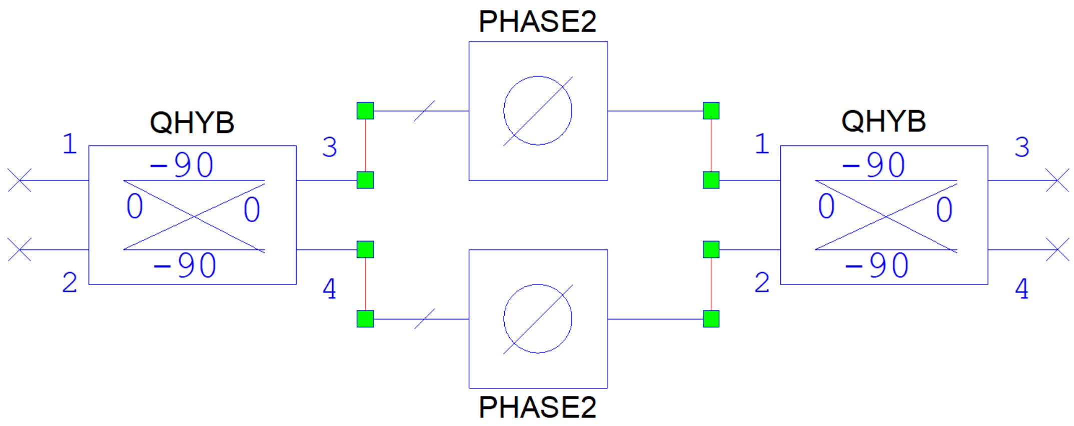

Figure S1. Basic MZI module used for the system simulation. The module was simulated as a combination of two 3 dB couplers (QHYB) and two phase shifters (PHASE), all centered to operate at 1 GHz. Notice that the QHYB coupling module is a typical symmetrical directional coupler as the one introduced in[2].

possible. To achieve this, we use pairs of the four-port coupler presented in Fig. (S3). The coupler implements the following (antisymetrical[2]) S-matrix

$$S = \begin{bmatrix} 0 & -\alpha & 0 & \beta \\ -\alpha & 0 & \beta & 0 \\ 0 & \beta & 0 & \alpha \\ \beta & 0 & \alpha & 0 \end{bmatrix} \tag{S1}$$

where α and β are the transmission and coupling coefficients, respectively. In all examples $|\beta|^2 = -20$ dB, i.e., 1% of the power is coupled to the output port while the rest ($|\alpha|^2 = 1 - |\beta|^2 = 99\%$) is directed to the system, while for the inversion of singular and rectangular cases the coupling coefficient was $|\beta|^2 = -30$ dB (0.1%). Notice that in both cases we utilized two couplers, allowing extra control over the extracted results and isolation of the output signal from the input signal, similar to[3].

1.3 Miller architecture

The basic modules can be combined to form the Miller architecture to perform matrix multiplication. With the addition of the feedback loop and coupling elements, linear equation solving can be achieved. This is depicted in Fig. (S4) for a 7×7 discretized system. Note that this architecture comprises of 28+7+28=63 MZI modules. In this particular example we utilized two different sets of feedback couplers, used for system inputs and outputs, respectively. Both sets had identical coupling/transmission coefficients.

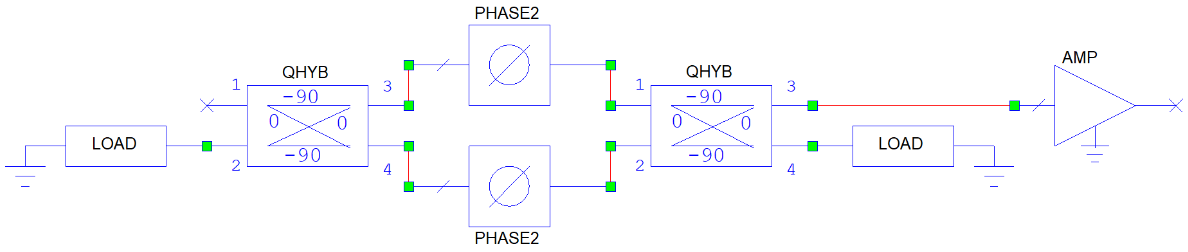

Figure S2. Basic MZI module followed by an amplification unit representing the multiplier module.

1.4 The DCM architecture

An alternative to the Miller architecture for matrix multiplication is the DCM architecture. Similar with the above, Fig. (S4) depicts the 5×5 equation solving system used to extract the presented results in the main text. For this system, a total of $5^2 = 25$ MZIs are used for the middle stage. Both the ingress and egress stages (Fig. (S5)) comprise a total of 5×4 MZIs with fixed phase shifts ratios. These devices are passive and are necessarily lossy. Both ingress and egress stages introduce a signal division of the order of $\alpha_{\text{in}} = \alpha_{\text{en}} = 1/\sqrt{5}$. The power losses associated with the ingress and egress stages are $-10\log(5^2)$, and can be compensated with an amplification stage (that is part of the main MZI module) that introduces a total of $10\log(5^2) = 13.97$ amplification to the system. Additionally, the feedback coupler introduces a α_{fc} loss to the system. Therefore, the DCM system can be described by the following equations

$$\mathbf{x} = \beta \mathbf{e}_{\text{in}} + \alpha_{\text{in}}\alpha_{\text{eg}}\alpha_{\text{fc}}\mathbf{K}\mathbf{x} \tag{S2}$$

and in this case the resulted inversion will yield to the following solution

$$\mathbf{x} = \left(\mathbf{I} - \alpha_{\text{in}}\alpha_{\text{eg}}\alpha_{\text{fc}}\mathbf{I} + \alpha_{\text{in}}\alpha_{\text{en}}\alpha_{\text{fc}}\mathbf{A}\mathbf{x}\right)^{-1}\mathbf{e}_{\text{in}} \tag{S3}$$

In the ideal case the transmission coefficients $\alpha_{\text{in}}\alpha_{\text{eg}}\alpha_{\text{fc}}$ can all be compensated by the amplification stage with gain g, hence the overall solution will be $\mathbf{x} \approx \mathbf{A}^{-1}\mathbf{e}_{\text{in}}$, assuming that the overall required amplification level is $g = 10\log(5^2/|\alpha_{\text{fc}}|^2)$.

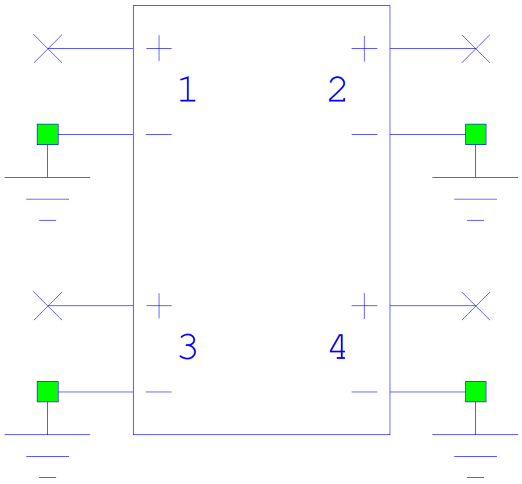

Figure S3. Module that is used for the feedback couplers, centered to operate at 1 GHz.

1.4.1 The ingress and egress stages

For the particular example of an 11×11 network, both the ingress and egress stages were simulated as a cascade of 10 MZI with fixed splitting ratios, i.e., the 1st MZI had a 1:10 balance, the 2nd 1:9, ..., and the 10th had 1:1 balance, designed so that if the cascade is illuminated with 1W of power, one would find 1/11 W on the outputs of each splitter. Both of these stages can be seen in Fig. (S6)

1.5 Results retrieval

From a system point of view, the solution (regardless of the architectures) can be retrieved by simply probing the input channels, just before the main entrance operator. In reality such measurement will disturb the actual result. For these reasons, the results are extracted using a second set of feedback couplers, as seen in Fig. (S4) and Fig. (S5), top-left corner. Assuming that the second set of couplers have the same coupling/transmission ratios as the first, then the extraction of the results is a straightforward task, similar to the one presented in[3]. Using a separate set of couplers for the input and output prevents the uncoupled input energy from overwhelming the output signal.

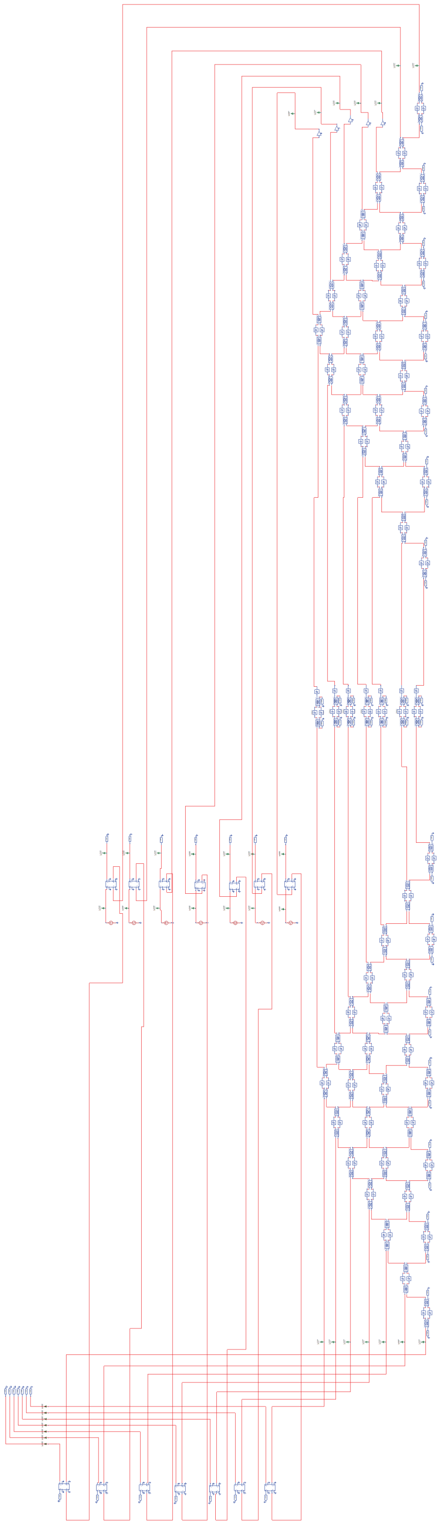

Figure S4. A schematic of the Miller architecture, centered to operate at 1 GHz. Note that in this scheme two feedback couplers were used, one for the input and one for the output of the system.

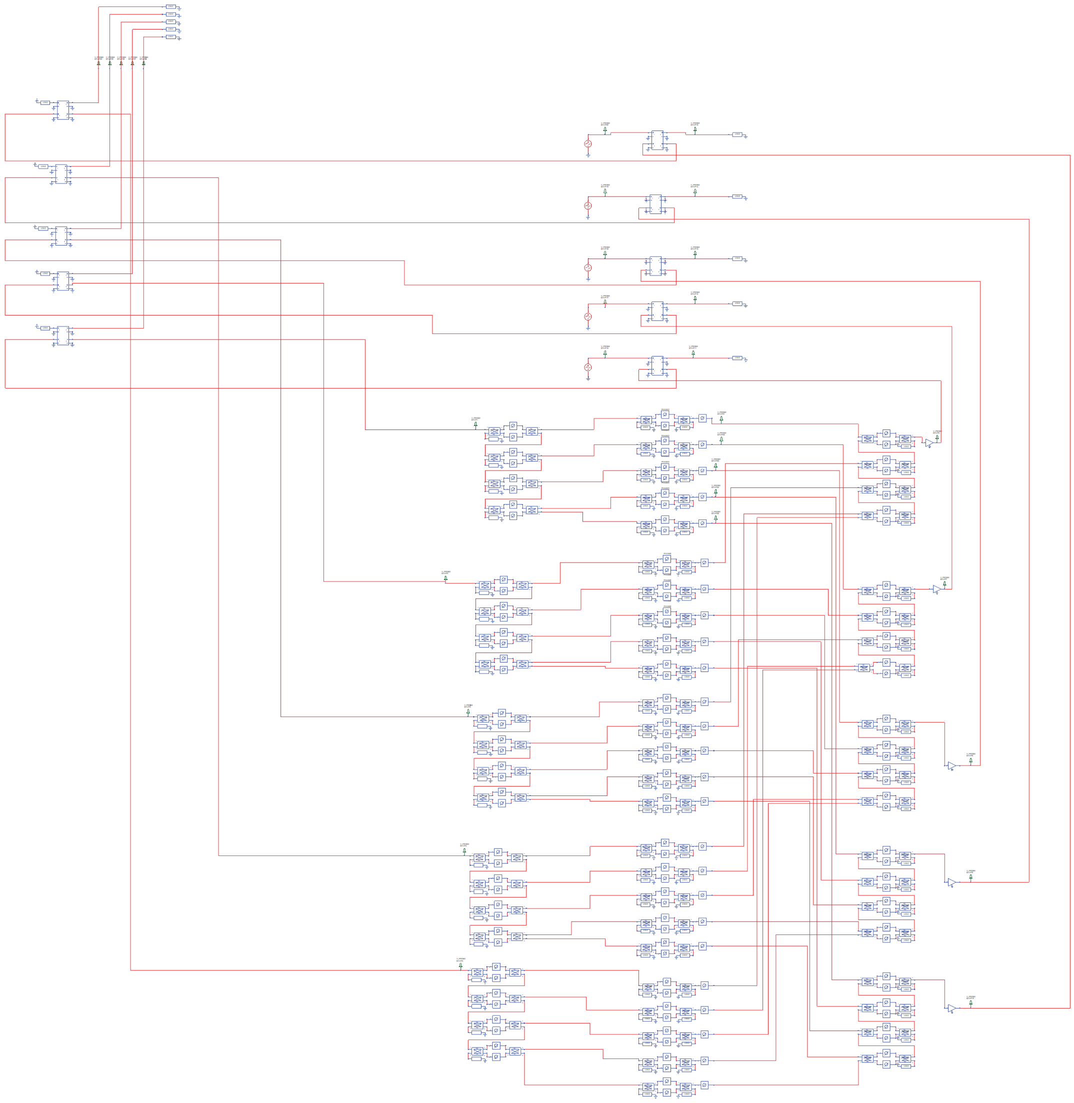

Figure S5. A schematic of the 5 × 5 DCM architecture all centered to operate at 1 GHz.

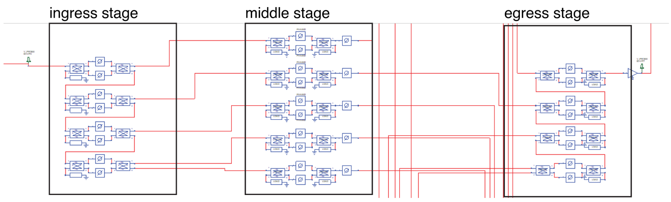

Figure S6. A detail of the the 5 × 5 DCM architecture depicting the the ingress, middle, and egress stages.

1.6 Statistical comparison of Miller and DCM architectures

Here we include an example where we performed a matrix inversion of a rectangular complex-valued 3x5 matrix with a series of tolerance/yield analyses for both architectures. In particular, we examine what is happening when we assume that the MZIs (phase-shifting elements) exhibit a normal distribution variation applied to the phase shifter and to the specific phase value. Note that for DCM architecture, both ingress and egress stages could be implemented using variable MZI components, but since the coupling ratios are fixed, we are assuming that these were implemented using fixed perfect splitters, hence we exclude them from our statistical analysis. Additionally, we did not include the effects of the amplifier noise, as this would confound the comparison between the two architectures.

First, let's examine the new complex-valued rectangular matrix. The randomly chosen matrix reads

$$A = \begin{bmatrix} 0.593-0.281i & 0.272+0.926i & 0.7-0.159i & -0.25-0.361i & 0.676+0.035i \\ -0.328+0.74i & -0.66-0.23i & -0.267-0.383i & -0.01-0.73i & 0.62+0.49i \\ 0.35+0.798i & -0.53-0.363i & -0.002-0.325i & 0.965-0.24i & -0.051+0.163i \end{bmatrix} \tag{S4}$$

For the matrix inversion, we use the same system-level simulation process used in the text. The numerical pseudoinverse of this matrix reads

$$A^{+} = \begin{bmatrix} 0.079-0.05i & -0.377-0.207i & 0.454-0.281i \\ 0.197-0.294i & -0.105+0.112i & 0.019+0.1i \\ 0.545+0.183i & -0.076+0.207i & 0.047+0.36i \\ 0.087+0.004i & -0.273+0.238i & 0.543-0.035i \\ 0.365-0.108i & 0.405-0.17i & -0.048+0.286i \end{bmatrix} \tag{S5}$$

Figure S7.S shows that both the DCM and Miller architectures successfully calculate the corresponding pseudoinverse of the matrix given above. To extract these values, we use the same settings of Fig. 4 (in the main text), i.e., couplers with a 30 dB coupling coefficient. Here, we used precisely the preconditioning shown in Eq. (8) and (12) found in the main text.

In particular, we have assumed that the kernel is

$$\mathbf{K} = \mathbf{I} - \alpha_\lambda \mathbf{A}^* \mathbf{A} \tag{S6}$$

with $\alpha_\lambda = 0.25$. Due to the rectangular nature of the matrix $\mathbf{A}$, the smallest eigenvalues of $\mathbf{A}^*\mathbf{A}$ are zero. Therefore, when performing the SVD of $\mathbf{I} - \alpha_\lambda \mathbf{A}^*\mathbf{A}$, as required in the Miller architecture, we find that the corresponding two larger singular values of the kernel are $s_1 = s_2 \approx 1$. Having eigenvalues that are very close to unity makes the convergence of the system very poor, yielding noise sensitivity that may lead to inaccurate results. For these reasons in the Miller architecture, since these two singular values can affect its convergence, we reduce these values to be $s_1 = s_2 = 0.5$. In other words, since we know the

spectral distribution of the system (due to the SVD) we tune the extreme eigenvalues of the kernel to be within the unit circle. As we will see, not only does this choice improve the accuracy of the results for the Miller architecture, but also it endows the Miller architecture with robust characteristics.

Next, we endeavor to reduce the sensitivity of the DCM. Obviously, we cannot do the same for DCM as was done for the Miller architecture since we do not have access to any kind of spectral information of the matrix – no a-priori mathematical operation is done to the kernel. Instead, we introduce a small perturbation to the kernel, i.e,

$$\mathbf{K} = \mathbf{I} - \alpha_\lambda \mathbf{A}^*\mathbf{A} - \delta\mathbf{I} \tag{S7}$$

where δ is a small value – these kinds of perturbations are typically encountered in Tikhonov-type regularization schemes (see Methods in text). In this sense, we reduce the sensitivity of DCM by introducing small reduction. Since for the case of rectangular/singular matrix $\mathbf{A}$, one or more of the eigenvalues is zero, this reduction will pull the eigenvalues of $\mathbf{I} - \alpha_\lambda \mathbf{A}^*\mathbf{A}$ slightly away from the rim of the unit circle.

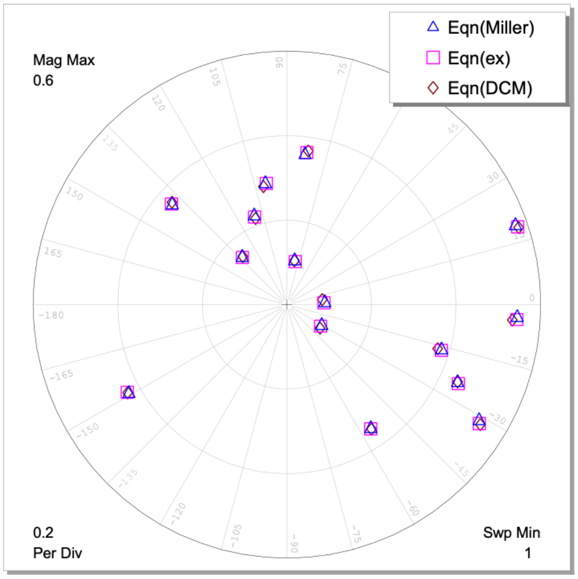

Figure S7. Comparison of the architectures for a matrix inversion complex-valued 3×5 matrix. The blue triangles denote inversion with the Miller architecture, the pink squares the exact pseudoinverse, and the brown rhombuses the inversion using the DCM architecture. The result was obtained in AWR Microwave Office®. Top. Note that for the Miller architecture, the first two singular values were selected to be $s_1 = 0.5$ and $s_2 = 0.5$, improving the accuracy and noise tolerance of the results.

Turning to the statistical analysis below (Figs. (S8)- (S11)), we performed the following two studies. In all figures we assumed that each phase shifting component exhibits a random variation

$$\theta_{var} = \theta_{\text{initial}} + 2\pi\mathcal{N}(\mu, \sigma^2) \tag{S8}$$

where $\mathcal{N}(\mu, \sigma^2)$ is the normal distribution with mean $\mu = 0$. In Figs. (S8) and (S9) the standard deviation is $\sigma = 0.01\%$. This translates to a total value variation of ± 0.0017(rad) or ± 0.1(deg) per element. Here we assumed a 3σ interval, i.e., $\pm 360 \times 3\sigma = 0.1$(deg). For this set of results the first two singular values are chosen to be $s_1 = s_2 = 0.95$ (Miller), while the perturbation term is $\delta = 0.02$ (DCM).

The results indicate that the Miller architecture has a better response to phase variations when compared to DCM. This result demonstrates that Miller's architecture is more robust and tolerant to noise. These features are mainly due to the SVD decomposition. DCM is slightly more sensitive to phase variations - in DCM, we need to use a gain stage that also amplifies the various effects of the overall system.

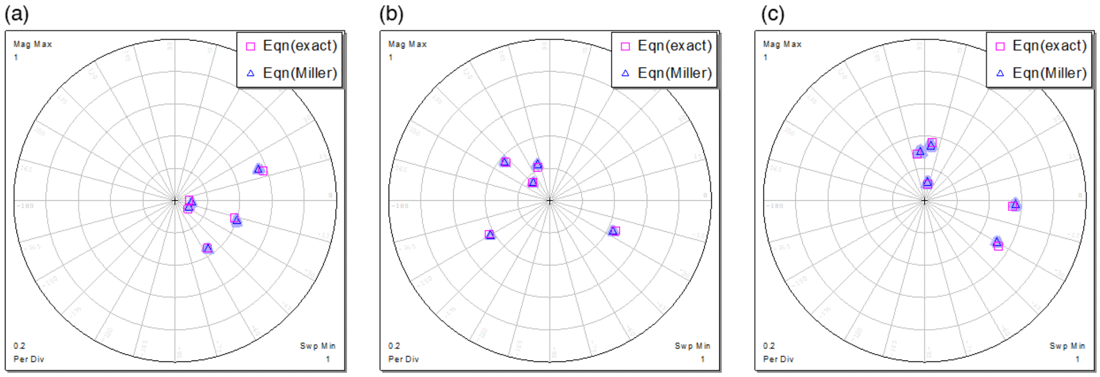

Figure S8. The Miller system used for inversion of the rectangular matrix for the case where the phase shifters of the MZI modules include noise with a normal distribution with a 0.01% standard deviation around the nominal values (case θ_{var}). The insets (a), (b), and (c) show each column of the inverted matrix. Notice that the two larger singular values (of the SVD) eigenvalues were chosen as $s_1 = 0.95$ and $s_2 = 0.95$.

The second statistical comparison, shown in Figs. (S10) and (S11), demonstrates the same distribution but only this time for $\sigma = 0.1\%$ (± 1(deg)). Similarly, Miller architecture demonstrates somewhat improved behavior when compared to DCM.

References

1. Cadence. AWR Microwave Office (2020).

2. Pozar, D. M. *Microwave engineering; 3rd ed.* (Wiley, Hoboken, NJ, 2005).

3. Estakhri, N. M., Edwards, B. & Engheta, N. Inverse-designed metastructures that solve equations. *Sci. (80-.).* **363**, 1333–1338, DOI: 10.1126/science.aaw2498 (2019).

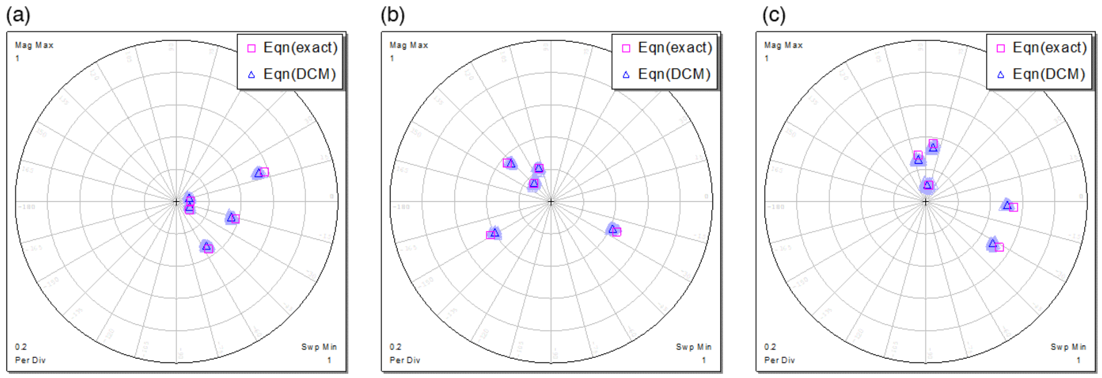

Figure S9. The DCM system used for inversion of the rectangular matrix for the case where the phase shifters of MZI modules exhibit a normal distribution with a 0.01% standard deviation around the nominal values (case θ_{var}) and $\delta = 0.02$. The insets (a), (b), and (c) show each column of the inverted matrix.

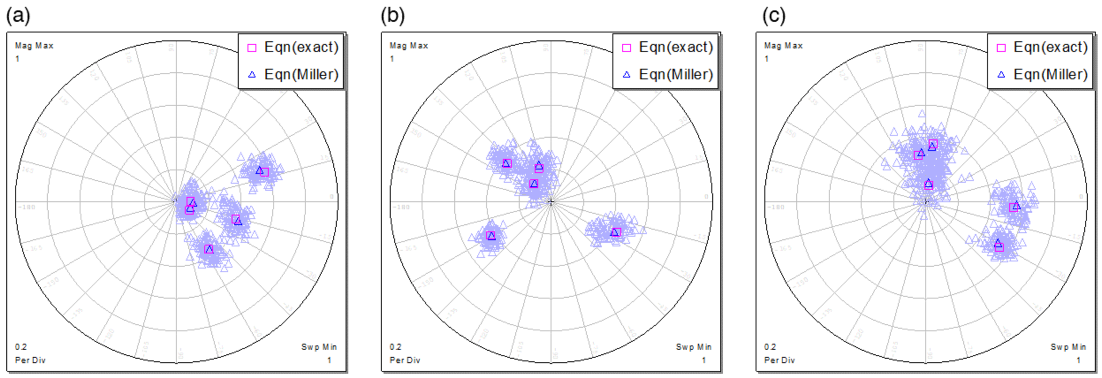

Figure S10. The Miller system used for inversion of the rectangular matrix for the case where the phase shifters of the MZI modules exhibit a normal distribution with a 0.1% standard deviation around the nominal values (case θ_{v2}). The insets (a), (b), and (c) show each column of the inverted matrix. Notice that the two larger singular values (of the SVD) eigenvalues were chosen to be $s_1 = 0.95$ and $s_2 = 0.95$.

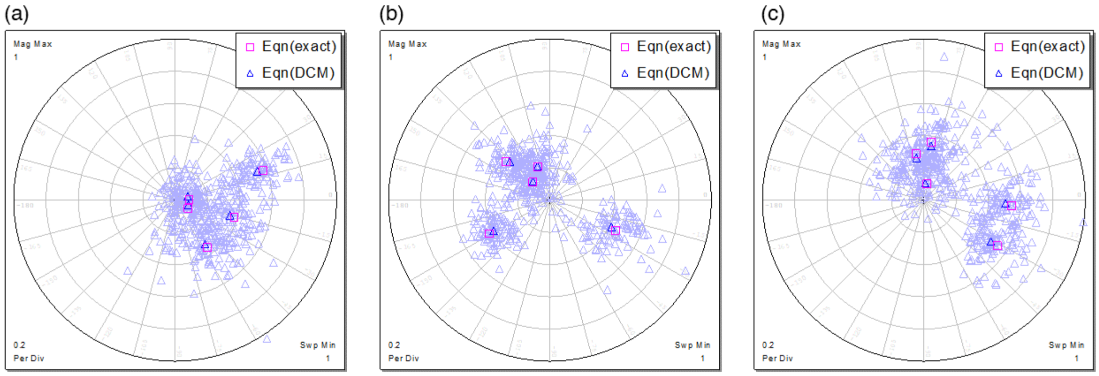

Figure S11. The DCM system used for inversion of the rectangular matrix for the case where the phase shifters of MZI modules exhibit a normal distribution with a 0.1% standard deviation around the nominal values (case θ_{var}) and $\delta = 0.02$. The insets (a), (b), and (c) show each column of the inverted matrix.
